# Topography hierarchy of biocompatible polyhydroxyalkanoate film†

**DOI:** 10.1039/d4ra03398a

**Published:** 2024-06-18

**Authors:** Fares D. E. Ghorabe, Aleksandr Aglikov, Alexander S. Novikov, Michael Nosonovsky, Galina A. Ryltseva, Alexey E. Dudaev, Natalia G. Menzianova, Ekaterina V. Skorb, Ekaterina I. Shishatskaya

**Affiliations:** a Infochemistry Scientific Center, ITMO University Lomonosova St. 9 191002 St. Petersburg Russia skorb@itmo.ru shishatskaya@inbox.ru; b Mechanical Engineering, University of Wisconsin-Milwaukee 3200 N Cramer St. Milwaukee WI 53211 USA nosonovs@uwm.edu; c School of Fundamental Biology and Biotechnology, Siberian Federal University Svobodnyi Av. 79 660041 Krasnoyarsk Russia; d Institute of Biophysics SB RAS, Federal Research Center “Krasnoyarsk Science Center SB RAS” Akademgorodok, 50/50 660036 Krasnoyarsk Russia

## Abstract

Polyhydroxyalkanoates (PHAs) are used for various biomedical applications due to their biocompatibility. Surface properties, such as surface roughness, are crucial for PHAs performance. Traditional parameters used for the characterization of surface roughness, such as *R*_a_, are often insufficient to capture the complex and hierarchical (multiscale) topography of PHA films. We measure the topography and surface roughness of thin PHA films with atomic force microscopy and analyze the topography data using several relatively novel data processing methods, including the calculation of autocorrelation functions, topological data analysis, and the distribution of minimum and maximum values of pixels over the topography data. The results provide details of multiscale and anisotropic surface properties that are crucial to PHAs biocompatibility but often overlooked by traditional topography analysis methods.

## Abstract 2

Synthesis and characterization process of the PHB granules and film.

## Graphical abstract

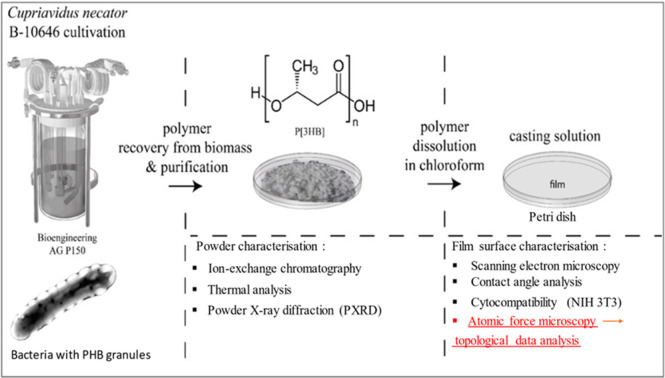

## 1. Introduction

Biodegradable polymeric materials are used in tissue engineering to produce highly porous volumetric structures, such as scaffolds, for the cell microenvironment that contribute to guided tissue regeneration. Polyhydroxyalkanoates (PHAs) of microbiological origin have great potential for synthesizing such scaffolds.^1–3^

PHAs are biocompatible and biodegradable polyesters that vary depending on the number of carbon atoms in the monomer. They can be divided into two main groups: the short-chain-length (scl-PHAs), which contain monomer units with 3–5 carbon atoms and the medium-chain-length (mcl-PHAs) with monomer units with 6–14 carbon atoms.^4,5^ The combination of monomers in the composition of the main chain makes it possible to control the physical and mechanical properties of PHA-based materials and to obtain scaffolds for bioengineering of different tissues with various biomechanical properties.^6^

Since PHAs are degraded to carbon dioxide and water without producing unwanted intermediate products, they can used for production of scaffolds for different tissues. They also maintain their shape for a long time and support the adhesion and proliferation of cells in various tissues, which makes them desirable for applications in biomedical engineering.^2,7–14^

The most well-studied and widespread representative of PHAs is poly(3-hydroxybutyrate) (P3HB). This polymer has good biocompatibility and biodegradability, and, while there are copolymer PHAs with characteristics superior to P3HB, for our goal, which is the surface roughness analysis of the casting PHA films to predict cellular reactions to implants made of these materials, P3HB was chosen to simplify analysis.^15–17^

The surface roughness of the material plays a key role in forming the body's response to implantation, having a direct effect on cell adhesion, the first stage in the emerging cell^18,19^ – biomaterial interactions.^20,21^ The control of interactions between cells and surfaces is an important aspect of materials bioengineering. An effective approach to this is to control the roughness parameters. The simplest conventionally three-dimensional system for evaluating cell–material interactions^22,23^ for structural polymeric materials is a film, which is usually obtained by pouring a polymer solution onto a template.^24–26^

The relief formed at the air-film interface during the evaporation of the solvent is stimulated by supramolecular processes that accompany crystallization, including self-organization and supramolecular self-assembly. The ability of PHAs to self-organize into hierarchical (multiscale) reliefs during the casting of solutions due to non-covalent bonds such as H-bonds, van-der Waals and hydrophobic interactions allows us to consider such film systems as the simplest prototype of a biomimetic polymer scaffold that has the topographic properties of a natural extracellular matrix.^27,28^ Atomic Force Microscopy (AFM) is a widely used technique to study different surfaces including polymers and to obtain their topography maps.^29,30^

The self-assembly often leads to multiscale or hierarchical surface features (*i.e.*, with small-scale roughness imposed on large-scale roughness), to self-affine or even to self-similar (fractal) structures. Note that self-similar and hierarchical structures are not the same. While self-similar structures do not have any characteristic length scale at all, hierarchical structures possess several length scales forming a hierarchy. The traditional surface roughness parameters, such as the mean roughness, *R*_a_, are often insufficient to characterize complex surfaces, which may possess non-Gaussian random roughness features (asperities) of different shapes, with different vertical and horizontal size scales, which may also be anisotropic, moreover, with different orientations of anisotropic features (such as scratches) at different scales. Novel methods of studying raw topographic data, such as topological data analysis (TDA), are currently actively developing.^31–33^ In this paper, we bring to your attention the study of a biodegradable polymer using TDA to search for self-similarity (invariance of different scales).

## 2. Materials and methods

### 2.1. Materials

Schlegel's mineral medium for the cultivation of bacteria served as the basic solution for growing cells. It was composed of disodium phosphate Na_2_HPO_4_·H_2_O (9.1 g L^−1^), monopotassium phosphate KH_2_PO_4_ (1.5 g L^−1^), magnesium sulfate MgSO_4_·H_2_O (0.2 g L^−1^), iron(iii) citrate FeC_6_H_5_O_7_·5H_2_O (0.025 g L^−1^), and urea CO(NH_2_)_2_ as a nitrogen source at the concentration of 1.0 g L^−1^.^34^ To stabilize the acidity level of the culture medium at 7.0 ± 0.1 pH, no adjustment was necessary due to urea acting as both a nitrogen source and pH stabilizer.

Hoagland's trace element solution was added at a rate of 3 mL L^−1^ to provide essential micronutrients for bacterial growth; it included specific concentrations of boric acid H_3_BO_3_, cobalt(ii) chloride CoCl_2_·6H_2_O, copper(ii) sulfate CuSO_4_·5H_2_O, manganese(ii) chloride MnCl_2_·4H_2_O, zinc(ii) sulfate ZnSO_4_·7H_2_O, sodium molybdate NaMoO_4_·2H_2_O, and nickel chloride NiCl_2_. All reagents used for the preparation of media had a purity of 99%.^35,36^

Glucose with a purity level of 98% served as the main carbon substrate and underwent sterilization *via* membrane filtration using Opticap XL300 Millipore Express SHC filters.

### 2.2. Microorganism description, culture medium and cultivation conditions

The inoculum was prepared by resuspending the museum culture maintained on agar medium. To prepare the museum culture, 1.0 L glass flasks were filled halfway with saline liquid medium containing glucose at an initial concentration of 5 to 10 g L^−1^. In this first phase, cells were cultivated for 25–30 hours.

The first-phase cell culture was transferred into larger (2–3 L) flasks, and cells were cultured for 30–35 h in the medium of the same composition but without nitrogen source and with a carbon substrate concentration of 10–15 g L^−1^. During cultivation, samples of culture medium were taken for analysis. Glucose concentration was determined spectrophotometrically at 490 nm by the glucose oxidase method using a Fotoglucoza kit (Impact Ltd, Moscow, Russia).

The inoculum was then cultivated in a 30 L fermenter (Bioengineering AG, Switzerland) in the pilot production (PP) line with a starting inoculate of (10–15 g L^−1^). The process of building up the seed material was carried out on a complete nutrient medium. The PP facility includes units for media and inoculum preparation, a unit for fermentation, and a unit for polymer extraction and purification. The PP fermentation unit has a steam generator (Biotron, South Korea) for sterilizing fermenters and connecting lines, a compressor (Remeza, Belarus) for air supply, a 30 L seed culture fermenter, a 150 L production fermenter (Bioengineering AG, Switzerland), an ultrafiltration unit (Vladisart, Russia) to concentrate the culture, and a unit for freeze-drying of the condensed bacterial suspension (LP10R ILSHIN C, South Korea). Fermenters were equipped with systems for monitoring pH level, foam level, temperature, pressure, and dissolved oxygen. The fermenters were controlled by BioScadaLab software in automatic mode.

To supply the feeding substrates, the fermenters were equipped with Bioengineering Peripex peristaltic pumps. The concentration of dissolved oxygen was maintained at DO 30%. The air supply control was carried out in a cascade mode (DO-air flow-mixer revolutions). During cultivation in a 30 L fermenter, the amount of air supplied per cultivation process varied from 0 to 5.5 NL min^−1^ (ANR – Atmosphere Normale de Reference), the speed varied from 500 to 1000 rpm. During cultivation in a 150 L fermenter, the amount of air supplied per cultivation process varied from 5 to 200 NL min^−1^, the speed varied from 300 to 750 rpm.^37^

### 2.3. P3HB biosynthesis, recovery, and production of films

P3HB was synthesized with the standard cultivation procedure of *Cupriavidus necator* B10646, capable of synthesizing polyhydroxyalkanoates at high yields in the Laboratory of New Materials Biotechnology, Siberian Federal University.^38–40^ The process of polymer synthesis was conducted in bioreactor system (P150, Bioengineering AG, Switzerland) with glucose as a carbon substrate. The polymer extraction was performed in two stages. Firstly, the biomass was processed with ethanol to remove lipids and fatty acids. Secondly, we make the polymer extraction with dichloromethane. The dichloromethane extracts were pooled and evaporated twice using an R/210 V rotary evaporator (Büchi, Flawil, Switzerland). Then, the polymer was precipitated with hexane. To purify the polymer, it was redissolved in chloroform several times and precipitated using hexane. The resulting polymer was dried at 40 °C.^41^

The P3HB film samples were prepared using the solution-cast method.^39^ P3HB was dissolved in chloroform to obtain a homogeneous solution (3%). The solution was poured onto defatted glass Petri dishes, followed by drying for 48 h in a vacuum desiccator (Labconco, Kansas City, Missouri, USA). LEGIONER EDM-25-0.001 electronic digital micrometer (Legioner, China) was used to measure the thickness of the films. Measurements were made in six zones. The obtained films were used for the analysis, cutting them into samples of appropriate sizes.

### 2.4. Characterization methods

The determination of the intracellular content and composition of the PHA polymer was carried out using gas chromatography mass spectrometry (GC-MS) of methyl esters of fatty acids. Methanolysis of the cell biomass was performed by boiling a 4.0–4.5 μg polymer sample under reflux condensers for 160 minutes in a mixture containing 1 mL chloroform, 0.85 mL methanol, and 0.15 mL concentrated sulfuric acid.

After completion of the methanolysis reaction, distilled water (1 mL) was added to the flask. The bottom chloroform layer obtained from this process was used for chromatographic analysis using a gas chromatograph-mass spectrometer equipped with a mass detector (7890A Agilent Technologies, U.S., model 5975C). This allowed for determination of both purity and composition of the polymer through analysis of methyl esters of fatty acids.^42,43^

To determine the content of the polymer in the resulting mass after precipitation, analysis was performed utilizing a gas chromatograph equipped with a chromatograph-mass spectrometer (7890A and 5975C respectively) manufactured by Agilent Technologies (U.S.). For purification purposes, the polymer underwent several cycles of dissolution in chloroform followed by precipitation using either isopropanol or hexane. Finally, drying of the resultant purified polymer occurred at 40 °C.^44^

Molecular weights and molecular weight distributions of PHA were examined with a gel permeation chromatograph (Agilent Technologies 1260 Infinity, U.S.) with a refractive index detector, using an Agilent PLgel Mixed-C column. Chloroform was the eluent. Calibration was done using polystyrene standards (Fluka, Switzerland, Germany).

PXRD structure analysis was performed to determine crystallinity employing a D8 ADVANCE X-ray powder diffractometer equipped with a copper cathode tube and VANTEC fast linear detector (Bruker, AXS, Germany). The degree of crystallinity (Cx) was calculated as a ratio of the total area of crystalline peaks to the total area of the radiogram (the crystalline + amorphous components). Calculations were done by using the Eva program of the diffractometer software^40,41^ PXRD analysis was carried out for both polymer powder and thin film.

All initial materials and blends were analyzed using FTIR spectroscopy. IR spectra were taken in the 400–4000 cm^−1^ range using a “NICOLET6700” FT-IR spectrometer (Thermo Scientific, U.S.) and a Smart Orbit accessory, by the attenuated total reflection (ATR) technique.

Thermal properties of P3HB were studied with a DSC-1 differential scanning calorimeter (Mettler Toledo). Samples of P3HB (4.0 ± 0.2 μg) were placed in aluminum crucibles and heated at 10 °C min^−1^. Thermal degradation of the samples was investigated using a TGA2 thermal analysis system (Mettler Toledo, Schwerzenbac, Switzerland). The crystallization temperature (*T*_c_), the melting point (*T*_melt_) and thermal decomposition temperature (*T*_degr_) were determined from peaks on thermograms, using the StarE software 11.0 (Mettler Toledo, Switzerland).

The surface microstructure of PHA films was studied using scanning electron microscopy (FE-SEM S 5500, Hitachi, Japan). Prior to microscopy, the samples were sputter coated with platinum thin film (≈few tens nanometers) using the Leica EM ACE200 (Leica, Vienna, Austria) to obtain a good-quality image.

Wettability of film and surface properties were studied with a Drop Shape Analyzer – DSA – 25E (Krüss, Germany) using the DSA-4 software for Windows. The Owens, Wendt, Rabel and Kaelble method was used to calculate surface free energy and its dispersion and polar components (mN m^−1^).

The biocompatibility of the P3HB films was investigated in a culture of mouse fibroblasts NIH 3T3 (ATCC, Manassas, VA, USA), which have been seeded on cut to the required size P3HB discs using a template in 96-well plates at a concentration of 20 × 10^3^ cells per cm^2^. Cells were cultured under standard conditions in DMEM supplemented with 10% fetal bovine serum and an antibiotic/antimycotic solution (Gibco, Invitrogen, Waltham, MA, USA) in an atmosphere of 5% CO_2_ at a temperature of 37 °C in a CO_2_ incubator (New Brunswick Scientific, Edison, NJ, USA). Fluorescent staining of nuclear DNA and cytoplasm was carried out using DAPI and FITC dyes, respectively (Sigma-Aldrich, St. Louis, MO, USA).

The roughness of the film surface was assessed utilizing atomic-force microscopy, AFM, in semi-contact mode. For this analysis, an NTERGRA ACADEMY AFM system (NT-MDT, Russia) was employed in normal conditions. The study was conducted using an NSG01 cantilever with a tip curvature radius ranging between 6 and 10 nm. To ensure accurate measurements, multiple sites with an area of 5 × 5 μm were examined on the obtained film. The scans were performed at a higher resolution of 512 pixels to capture finer details. To enhance data quality and correct any artifacts present in the scans, Gwyddion software was utilized for post-processing.^45^

Cytocompatibility of P3HB cast films were assessed in NIH 3T3 mice fibroblasts culture. Cells were seeded into the wells of a 96-well culture plate with pre-placed polymer films at a concentration of 0.01 × 10^6^, in 100 μL of Dulbecco's Modified Eagle Medium (DMEM) (Sigma) with the addition of 10% fetal calf serum (HyClone™) and an antibiotic–antimycotic (Sigma). Cultivation was carried out in a humid atmosphere according to a standard protocol.^17^ Experiment was conducted for up to three days, then the cells were stained with DAPI (4′,6-diamidino-2-phenylindole) – blue fluorescent DNA dye, to contrast nuclei, and FITC (fluorescein 5-isothyocyanate) – green dye for cytoplasm.

### 2.5. TDA analysis

The average surface roughness, *R*_a_, of a randomly rough profile *z*(*x*) provides the vertical scale length (height) of largest surface asperities. However, no information about their horizontal length can be obtained from *R*_a_. To characterize the horizontal size of roughness details, the autocorrelation function (ACF) is often used. The ACF is constructed by averaging the correlation between two profile points separated by a given distance *τ*. Closer points tend to correlate more likely than remote points, consequently, the ACF decays as a function of distance, 
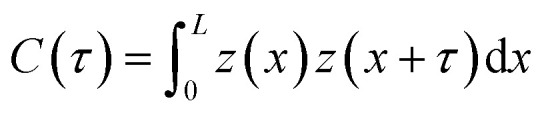
. The decay of the ACF to 10% of the original value (referred to as the correlation length β*, such that 
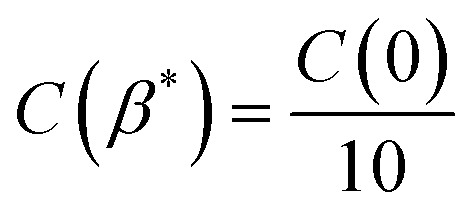
) characterizes the horizontal size of roughness details.

In addition, statistics of maxima and minima locations in 3 × 3 pixel patches can be used to characterize horizontal scale of surface roughness details on the scale of the pixel size, as well as roughness anisotropy. In many cases, the two methods, ACF and the statistics of 3 × 3 pixel patches, *p*_*ij*_, provide different results, which may be interpreted as imposing of a small-scale surface roughness (with the typical size comparable with the pixel size) on larger scale roughness.

The analysis of the statistical distribution of minima and maxima in 3 × 3 was suggested by ref. 33. Information about the horizontal size scale of surface roughness details (asperities), *L*, can be obtained from this distribution.

The length of roughness details can be estimated by eqn (1):1
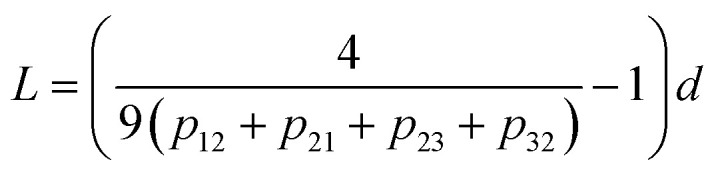
where *d* is the size of pixels. The distribution also provides information about the anisotropy of surface roughness at the scale of the pixel size, if two components are considered separately (eqn (2) and (3)).2
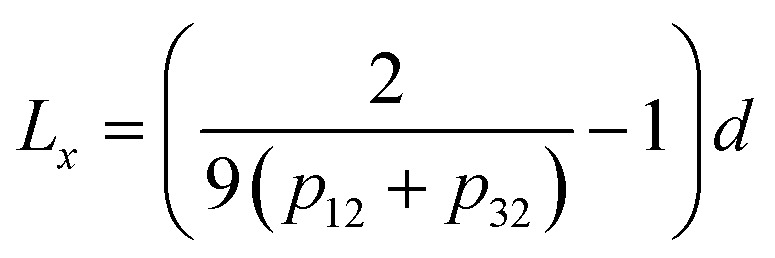
3
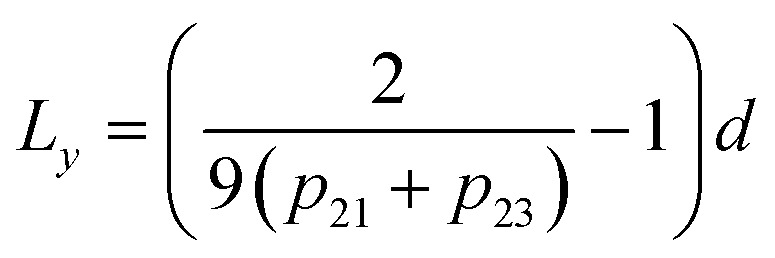


Besides the ACF, the so-called barcodes obtained by the Topological Data Analysis (TDA) are used to analyze anisotropy of surface roughness at different scale lengths. The intervals of the appearance and disappearance of features are plotted as columns on the barcode diagram with longer bars representing invariants that are resistant to noise.

Calculations are generated using the Python interface with the special TDA (Ripser) and standard libraries. Surface profile data in the form of a square matrix (256 × 256) were used as input for barcode generation, as described in ref. 32. The original matrix was divided into 3 × 3 fragments, which were used to create a filtered simplicial complex. We used Vietoris–Rips filtering to generate a simplicial complex. In a simplicial complex, data points form a topological space. Different colors of barcodes correspond to different topological invariants. Thus, the topological invariant H_0_ represents the entire structure without holes. The topological invariant H_1_ is a 1D hole (*i.e.*, a cylindrical structure) and H_2_ represents the 2D empty void (surrounded by a surface). The persistence diagrams and barcodes have been also successfully used for “data multiplication” required for the application of Machine Learning clustering algorithms.

## 3. Results

**Fig. 1:**
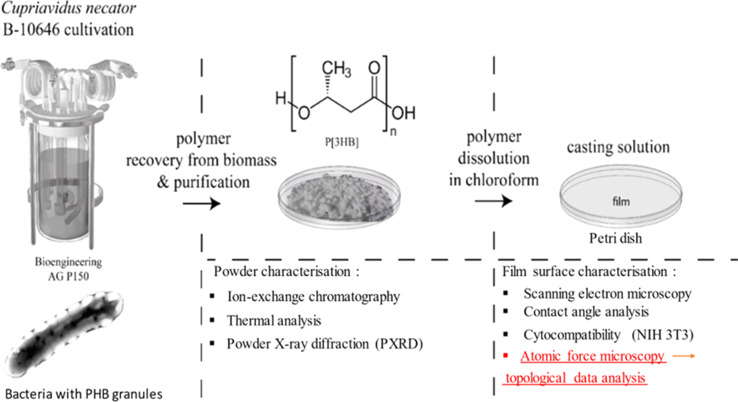
Production and characterization process of PHB granules and film.

**Fig. 2:**
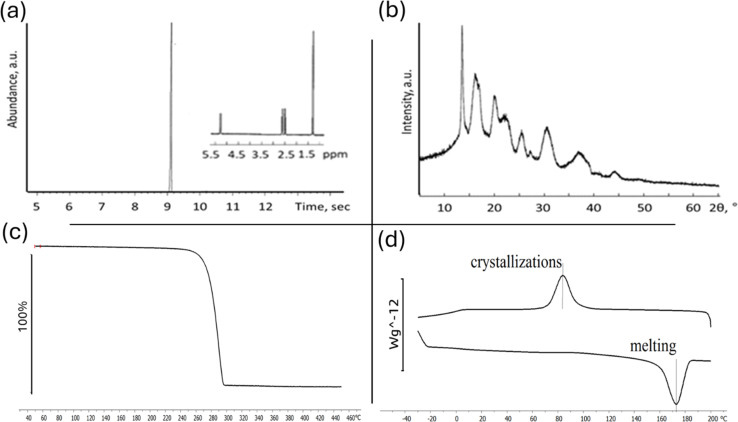
Comprehensive characterization of P3HB chemical analysis *via* ion chromatography (a), crystal structure PXRD (b), and thermal properties analysis (c) and (d).

The production and characterization processes are explained in Fig. 1. The left part of the scheme illustrates the production of PHB granules inside the bacterial cells in the fermenter. It starts with growing the strain in flasks using batch culture to activate it, followed by scaling up to pilot-scale feed batch cultivation. Then, biomass recovery and purification are performed to obtain polymer powder. The formed powder is shown in Fig. 2a. Resulting polymer powder is characterized using ion exchange chromatography, thermal analysis, X-ray diffraction. To obtain P3HB film, the polymer powder is dissolved in chloroform. The obtained film is characterized using AFM, contact angle analysis, cytocompatibility tests, and AFM scans for Topographic Data Analysis (TDA). Further details can be found in the Discussion section.

By culture *Cupriavidus necator* B-10646 we synthesized a homopolymer of 3-hydroxybutyric acid. P3HB is an isotactic polyester consisting of regular, identically oriented (“head-to-tail”) successive units of d-β-hydroxybutyric acid: [–O–CH(CH_3_)–CH_2_–CO–].^32^ The ion chromatogram with mass spectrum of the poly(3-hydroxybutyrate) powder is shown in Fig. 2b.

One crucial characteristic of polymeric materials is their degree of crystallinity, which represents the ratio between ordered (crystalline) and disordered (amorphous) phases. The ability of PHA to crystallize is determined by its internal chain properties and can be characterized by the crystallization temperature (*T*_c_). X-ray diffraction analysis reveals that the 3-hydroxybutyric acid homopolymer has a dominant crystalline phase over the amorphous phase (>70% Cx),^46^ as shown in Fig. 2c.

Temperature characteristics and crystallizability in their native state are significant parameters for P3HB since they influence its thermomechanical properties and processing possibilities into melt-based products (Fig. 2d). For P3HB, typical values include a crystallization range (*T*_c_) between −85 °C and −117 °C. The original polymer sample without prior exposure to heat melted within a range from 150 °C to 190 °C. The actual melting point (*T*_melt_) was found at approximately 162 °C with a sharp melting peak observed at around 179 °C (Fig. 2d). This peak exhibited high intensity and narrowness along with a small low-temperature tail. The thermal degradation range (*T*_degr_) was determined to be between 275 °C and 295 °C.

**Fig. 3:**
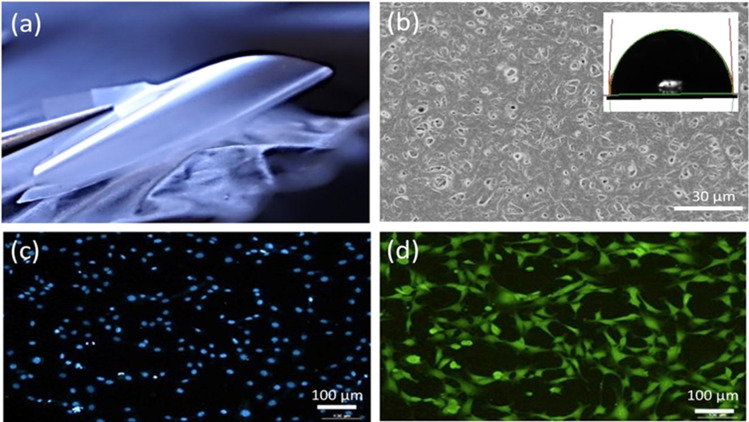
Surface characterization of P3HB film. (a) Photo of the P3HB film shows the flexibility and transparency of the film. (b) SEM of the P3HB film. Inset shows the water drop on the surface of the film and contact angle of 92°. (c) and (d) Fluorescent microscopy of P3HB films with NIH 3T3, DAPI and FITC.

Fig. 3a provide an appearance of P3HB polymer film. The cast films were thin, smooth, and practically transparent. Film thickness was 21.3 ± 2.5 μm.

The scanning electron microscopy (SEM) images presented in Fig. 3b provide surface morphology of the P3HB sample. The analysis reveals that the sample exhibits a well-developed surface structure, characterized by numerous pores distributed throughout. Most of these pores fall within the size range of 0.03 to 1.00 μm, with an average pore size measuring approximately 0.077 μm. The formation of pores in cast PHA films during solvent evaporation occurs due to polymer crystallization.

Furthermore, the hydrophilic/hydrophobic nature of the surface is evident from the water contact angle measurements depicted in Fig. 3b as an inset. The recorded contact angle value for water on this surface was found to be 92°, indicating a moderately hydrophobic behavior. In addition, quantitative analysis of surface energy revealed values for total surface energy, dispersed component, and polar component as follows: 30.8 mN m^−1^, 28.6 mN m^−1^, and 2.3 mN m^−1^.

Cultivation of fibroblasts on P3HB samples demonstrated good adhesion properties of the films and absence of cytotoxicity. The cells maintained high viability and were evenly distributed over the surface of the samples (Fig. 3c and d).

## 4. Discussion

**Fig. 4:**
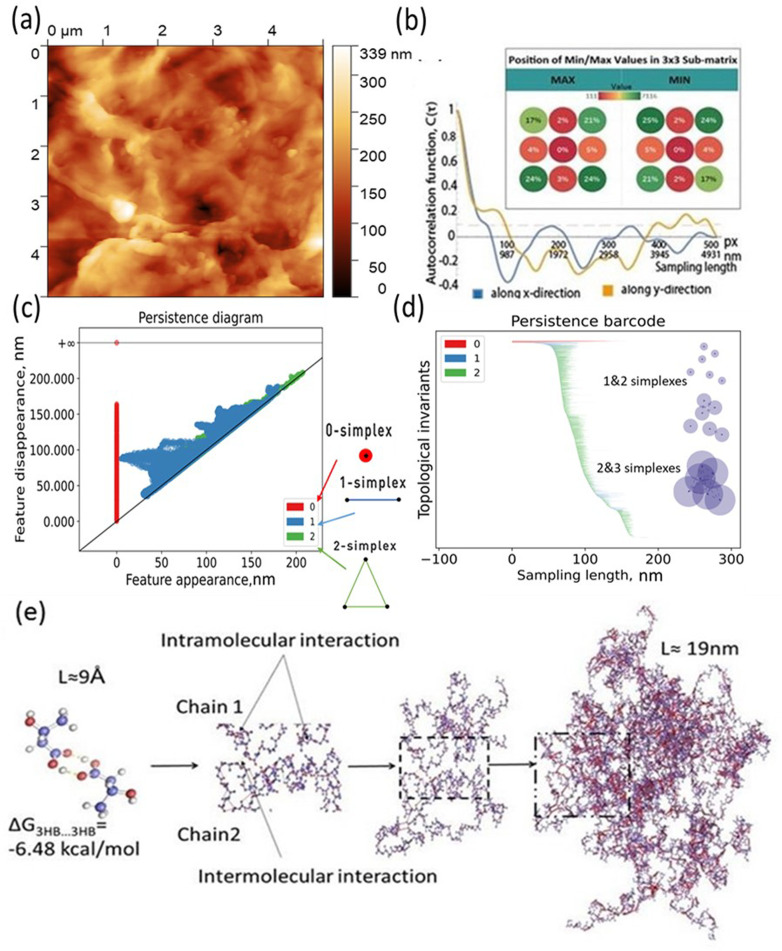
(a)–(d) AFM images and corresponding autocorrelation functions and persistence barcodes for the samples. (e) Schematic showing the hierarchical topography evolution of P3HB polymer chains: from 19 nm random order of 20 polymer chains to 9 Å P3HB monomer scale, and the inter–intra molecular interactions between polymers and monomers.

The main idea of the paper is to provide new barcode analysis for the AFM of P3HB films and point the sized of simplexes *vs.* molecular organization of polymer film Fig. 4.

To conduct further surface roughness analysis, the data topology method was used.^31–33^ The original 256 × 256 matrices were divided into 3 × 3 submatrices or patches (58 nm × 58 nm and 116 nm × 116 nm) and persistence diagrams and barcodes were generated.

The first topological invariant, H_0_ is persistent over the entire proximity radius range indicating to the connected component. The second topological invariant, H_1_, corresponds to 2D holes in data and it is significant at the resolution length corresponding to the size scale of the profile approximately in the range of 5–15 nm. The third topological invariant, H_2_, corresponds to 3D voids in the datasets and can be interpreted as the trace of the anisotropy of the profile. Its typical size is above 10 nm and the characteristic scale increases. Molecular weights (weight-average, *M*_w_, and number-average, *M*_*n*_) and polydispersity (*Đ* = *M*_w_/*M*_*n*_) were determined. *M*_w_ = 920 kDa, *M*_*n*_ = 368 kDa, *Đ* = 2.5.

The results for minima and maxima distribution are shown in Fig. 4b. For example, for pixel size *d* = 5 μm/512 = 9.77 nm, and the data shown, one can calculate *L* = 21.2 nm, *L*_*x*_ = 33.9 nm, and *L*_*y*_ = 14.4 nm.

On the other hand, the decay of the AFM to 10% of the initial value provides the values of the autocorrelation length as 
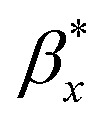
 =562.2 nm and 
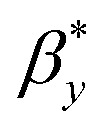
 = 937.2 nm (Fig. 4b), which is more than an order of magnitude larger than the values of *L*_*x*_ and *L*_*y*_. This shows that the nanoroughness with the characteristic length of dozens of nm imposed upon the larger roughness details with the characteristic length of hundreds of nm. The persistence diagrams (Fig. 4c) and barcodes (Fig. 4d) show the prevalence of H_1_ at the length scales from dozens to 175 nm. The second topological invariant, H_1_, corresponds to 2D holes in data and can be interpreted as corresponding to the primary circle due to the gradual change of the height. The topological invariant H_2_ corresponds to 3D voids in the datasets and can be interpreted as the trace of the anisotropy of the profile. Its typical size is above 25 nm. This is consistent with *L*_*x*_ = 33.9 nm, and *L*_*y*_ = 14.4 nm, which showed anisotropy at the scale of dozens of nanometers.

The topography hierarchy of P3HB, from monomers to oligomers and polymers, involves the arrangement of chains and interactions between them at different length scales. At the nanometer scale, P3HB monomers polymerize through esterification reactions, forming linear chains with repeating units. These chains can further aggregate into small clusters or crystalline domains due to intermolecular forces such as van der Waals interactions and hydrogen bonding. As the chain length increases, these aggregates become more pronounced Fig. 4e.

To evaluate the thermodynamic favorability for the hydrophobic/hydrophobic interaction between PHB monomers, we performed quantum chemical calculations using density functional theory (DFT). The RI BP86 level of theory was employed for these calculations. Further details can be found in the ESI.† The results of calculations revealed that self-assembly pf a functional monomeric unit of associate 3hb/3hb *via* hydrogen bonding O–H⋯O is thermodynamically favorable by 6.48 kcal mol^−1^ in terms of Gibbs free energies.

Moving up in scale to the micrometer range, P3HB oligomers consist of a few repeating units and exhibit greater flexibility compared to shorter chains. The interaction between neighboring oligomer segments is primarily governed by weak noncovalent forces like hydrophobic interactions.

The aggregation behavior becomes more evident at this level, leading to self-assembled structures such as fibrils or lamellae within individual domains. At higher scales in the micrometer range, P3HB polymers form large-scale structures due to interchain interactions involving both crystalline and amorphous regions. Crystalline regions exhibit ordered packing arrangements facilitated by strong intrachain bonds while amorphous regions possess irregular conformations influenced by chain entanglements and defects.

## 5. Conclusions

For the first time TDA analysis is applied to biocompatible and well-characterized by other methods PHA films and connected to the calculated molecular structure. It is pointed based on the DFT calculation the thermodynamic ability for the formation of hydrophobic bonding for the monomer units. The scales for molecular organization are shown. It is very prospective to teach the TDA in following how to connect the barcodes with the molecular computation. Self-similar structures do not have any characteristic length scale at all, the hierarchical structures possess several length scales forming a hierarchy. The traditional surface roughness parameters, such as the mean roughness, *R*_a_, are often insufficient to characterize complex surfaces, which may possess non-Gaussian random roughness features (asperities) of different shape, with different vertical and horizontal size scales, which may also be anisotropic, moreover, with different orientations of anisotropic features (such as scratches) at different scales. Novel methods of studying raw topographic data, such as TDA, are currently actively developing and very prospective in the area of biopolymers.

## Data availability

The data supporting this article have been included as part of the ESI.†

## Author contributions

Fares D. E. Ghorabe: investigation, methodology, validation, writing – original draft. Aleksandr Aglikov: investigation, methodology, validation, writing – original draft, project administration. Alexander S. Novikov: methodology, validation. Michael Nosonovsky: conceptualization, writing – review & editing. Galina A. Ryltseva: investigation, validation, writing – original draft. Alexey E. Dudaev: investigation, validation, writing – original draft. Natalia G. Menzianova: investigation. Ekaterina V. Skorb: funding acquisition, supervision. Ekaterina I. Shishatskaya: conceptualization, supervision.

## Conflicts of interest

The authors declare that they have no known competing financial interests or personal relationships that could have appeared to influence the work reported in this paper.

## Supplementary Material

RA-014-D4RA03398A-s001
