# Supplementary material for: Topography hierarchy of biocompatible polyhydroxyalkanoate film
Source: RSC Adv. 2024 Jun 18;14(27):19603–11. doi: 10.1039/d4ra03398a (PMC11184939; doi:10.1039/d4ra03398a)
Supplement: RA-014-D4RA03398A-s001 [file RA-014-D4RA03398A-s001.pdf]

Electronic Supplementary Material (ESI) for RSC Advances.
This journal is © The Royal Society of Chemistry 2024

Topography hierarchy of biocompatible polyhydroxyalkanoate films

Fares D.E. Ghorabe [1], Aleksandr Aglikov [1], Alexander S. Novikov [1], Michael Nosonovsky [2*], Galina A. Ryltseva [3], Alexey E. Dudayev [3,4], Natalia G. Menzianova [2], Ekaterina V. Skorb [1] and Ekaterina I. Shishatskaya [1]

1 Infochemistry Scientific Center, ITMO University, Lomonosova St. 9, 191002 St. Petersburg, Russia; skorb@itmo.ru

2 Depertment of Mechanical Engineering, University of Wisconsin-Milwaukee, 3200 N Cramer St., Milwaukee, WI 53211, USA, Email: nosonovs@uwm.edu

3 School of Fundamental Biology and Biotechnology, Siberian Federal University, Svobodnyi Av. 79, 660041 Krasnoyarsk, Russia; alex15-96@mail.ru, shishatskaya@inbox.ru

4 Institute of Biophysics SB RAS, Federal Research Center "Krasnoyarsk Science Center SB RAS", Akademgorodok, 50/50, 660036 Krasnoyarsk, Russia

* Correspondence: nosonovs@uwm.edu

Supporting Information

Computational details: In this study, we employed the ORCA software package to perform density functional theory (DFT) calculations. The chosen functional was RI BP86 with def2-SVP basis set for the molecular system under investigation. The def2/J auxiliary basis set and RIJCOSX approximation were utilized to enhance computational efficiency. We further incorporated D3BJ dispersion correction to account for van der Waals interactions. The initial step involved geometry optimization using a CPCM solvation model in chloroform solvent. We chose chloroform as the solvent because it was used in our experiments. The convergence criteria for SCF procedure were very tight. For both optimization and frequency calculations, we employed a conjugate gradient algorithm as the Z-matrix solver with a tolerance of 1e-6, ensuring accurate determination of nuclear coordinates and vibrational frequencies. To efficient convergence during SCF iterations, a maximum of 500 iterations was allowed. To balance computational resources utilization while maintaining accuracy, we limited the maximum memory usage (%maxcore) to 5000 MB throughout our simulations. Parallelization was implemented on 24 processors (%pal nprocs). The resulting optimized structure obtained from the previous step was then used as input in subsequent frequency calculations ("%base"opt" *xyzfile 0 1 opt.xyz"). This additional calculation aimed at obtaining vibrational frequencies and corresponding intensities for further analysis of all optimized model structures to prove the location of correct minima on the potential energy surface (no imaginary frequencies) and to estimate the thermodynamic parameters, the latter being calculated at 25 °C. The Cartesian atomic coordinates for optimized equilibrium model structures are presented in Table S1 and Table S2.

Table S1. Calculated total electronic energies, enthalpies, Gibbs free energies (in Hartree), and entropies (in cal/mol•K) for optimized equilibrium model structures (E, H, G, and S, respectively)

Model structure	E	H	G	S
3HB	-240136.96	-240056.43	-240082.35	-39.22
3HB/3HB	-480293.67	-480131.96	-480171.17	-39.21

Table S2. Cartesian atomic coordinates for optimized equilibrium model structures.

Atom	X	Y	Z
3HB			
C	-1.237052717	0.707272108	0.391238224
C	0.164444188	0.634137011	-0.217495826
C	1.157123413	-0.149623785	0.611260394
H	0.567722185	1.650240537	-0.412080635
H	0.100117362	0.139560606	-1.211745162
O	-2.07597348	1.24077516	-0.636928118
H	-2.979099066	1.292568045	-0.269346758
O	0.882480065	-0.894950288	1.540029116
O	2.427062135	0.04862862	0.186704319
H	3.005842483	-0.515160113	0.749603043
C	-1.286657339	1.562588131	1.659534185
H	-0.964325486	2.600856143	1.436452683
H	-0.629013013	1.142662844	2.446435705
H	-2.319044839	1.597491082	2.065762284
H	-1.546855892	-0.334036101	0.650396546
3HB/3HB			
C	-6.151503986	9.963720965	0.178484544
C	-5.661220758	9.023377843	-0.92505784
C	-5.642204597	7.561753245	-0.548384431
H	-4.653293613	9.318057634	-1.283471139
H	-6.337579648	9.121704595	-1.803191219
O	-6.330439301	11.23389795	-0.454373031
H	-6.64296903	11.85317917	0.232768018
O	-6.321385054	7.115587592	0.398209491
O	-4.87849212	6.816700921	-1.315287799
H	-4.941879637	5.813890055	-1.032422615
C	-5.176734061	10.05122922	1.355161156
H	-4.191169347	10.42807519	1.01107331
H	-5.035812841	9.060282747	1.83161027
H	-5.564071882	10.7445249	2.13062258
H	-7.1274212	9.56900169	0.550358101
H	-7.990872159	2.968787825	-0.960583538
H	-7.282050216	1.38367773	-1.443325
H	-8.848487373	1.45128498	-0.559718952

C	-7.843837565	1.914543006	-0.647424483
O	-4.978505954	4.335077319	-0.676175247
H	-6.347245504	5.641047087	0.766385207
O	-6.400789624	4.639865706	1.054941512
C	-5.655944478	3.889973458	0.269937018
C	-7.096302362	1.82562301	0.685068889
H	-5.0815852	1.876401875	-0.153986177
C	-5.679109088	2.41836713	0.60378538
H	-7.661835526	2.402791005	1.455909278
H	-7.805366176	0.066417032	1.145540222
O	-6.918061419	0.472132773	1.103525076
H	-5.191450282	2.280556345	1.592401419
